# Supplementary material for: Reconciling Mining with the Conservation of Cave Biodiversity: A Quantitative Baseline to Help Establish Conservation Priorities
Source: PLoS One. 2016 Dec 20;11(12):e0168348. doi: 10.1371/journal.pone.0168348 (PMC5173368; doi:10.1371/journal.pone.0168348)
Supplement: S1 Dataset — (ZIP) [file pone.0168348.s002.zip › Taxa/Serra Norte/SN_2013/N4E/Lista N4E-20.pdf]

CAVIDADE N4E-0020

Classe	Ordem	Fam/Outros	Gên/Outros	Espécie	S-U	U-S	Zon.
Arachnida	Acari	Trombiculidae		sp.39		X	E
Arachnida	Araneae	Ctenidae	*Nothroctenus*	*marshi*		2	E/P
Arachnida	Araneae	Drymusidae		jovem		X	E
Arachnida	Araneae	Filistatidae		sp.1	X	X	P
Arachnida	Araneae	Ochyroceratidae	*Ochyrocera*	sp.2		X	E
Arachnida	Araneae	Ochyroceratidae	*Speocera*	sp.1		X	E
Arachnida	Araneae	Pholcidae	*Mesabolivar*	sp.1	X	X	E
Arachnida	Araneae	Pholcidae	*Metagonia*	sp.	X		
Arachnida	Araneae	Pholcidae	*Modisimus*	sp.1		X	E
Arachnida	Araneae	Prodidomidae	*Lygromma*	sp.1		X	P
Arachnida	Araneae	Salticidae		jovem		X	E
Arachnida	Araneae	Scytodidae	*Scytodes*	sp.	X		
Arachnida	Araneae	Segestriidae	*Ariadna*	sp.	X		
Arachnida	Araneae	Tetragnathidae	*Leucauge*	sp.	X		
Arachnida	Araneae	Theraphosidae		sp.	X		
Arachnida	Araneae	Theridiosomatidae	*Plato*	sp.1		X	E/P
Arachnida	Opiliones	Stygnidae	*Protimesius*	*gracilis*		1	P
Arachnida	Pseudoscorpiones	Chernetidae	*Spelaeochernes*	sp.1		X	E
Arachnida	Pseudoscorpiones	Chthoniidae	*Pseudochthonius*	sp.1		X	P
Arachnida	Pseudoscorpiones	Olpiidae		jovem	X	X	E
Arachnida	Scorpiones	Buthidae	*Ananteris*	jovem		1	E
Diplopoda	Polydesmida	Pyrgodesmidae		sp.1		X	E
Diplopoda	Spirostreptida	Pseudonannolenidae	*Pseudonannolene*	sp.1	X		
Entognatha	Collembola	Entomobryidae		sp.9		X	E
Entognatha	Collembola	Paronellidae		sp.1		X	E/P
Entognatha	Diplura	Campodeidae		sp.1	X	X	E/P
Entognatha	Diplura	Projapygidae		sp.1	X		
Insecta	Blattodea	Blattellidae		jovem	1	2	E
Insecta	Coleoptera	Elateridae		jovem	X		
Insecta	Coleoptera	Tenebrionidae	Alleculinae	jovem	X	X	E
Insecta	Diptera	Cecidomyiidae	Cecidomyiinae	sp.	X	X	E
Insecta	Diptera	Psychodidae	Phlebotominae	sp.		X	E
Insecta	Diptera	Sciaridae		sp.		X	E
Insecta	Diptera	Tipulidae	Tipulinae	sp.	X	X	E
Insecta	Heteroptera	Cydnidae		jovem		X	E
Insecta	Heteroptera	Reduviidae	Emesinae	sp.		1	E
Insecta	Heteroptera	Reduviidae	*Zelurus*	sp.	X	4	E
Insecta	Homoptera	Cixiidae		jovem	X	X	E/P
Insecta	Hymenoptera	Formicidae	*Brachymyrmex*	sp.1		X	E
Insecta	Hymenoptera	Formicidae	*Camponotus*	aff. *bidens*		X	E
Insecta	Hymenoptera	Formicidae	*Camponotus*	sp.1		X	E
Insecta	Hymenoptera	Formicidae	*Camponotus*	sp.2		X	E
Insecta	Hymenoptera	Formicidae	*Gnamptogenys*	sp.1		X	E
Insecta	Hymenoptera	Formicidae	Myrmicinae	sp.1		X	E
Insecta	Hymenoptera	Formicidae	*Odontomachus*	sp.2		X	E
Insecta	Hymenoptera	Formicidae	*Paratrechina*	sp.2		X	E
Insecta	Hymenoptera	Formicidae	*Pheidole*	sp.	X		
Insecta	Hymenoptera	Formicidae	*Pheidole*	sp.1		X	E
Insecta	Hymenoptera	Formicidae	*Pheidole*	sp.2		X	P
Insecta	Hymenoptera	Sphecidae		sp.	X		
Insecta	Isoptera	Termitidae	*Nasutitermes*	sp.	X	X	E
Insecta	Lepidoptera	Noctuoidea		sp.5		1	E
Insecta	Lepidoptera	Tineoidea		sp.	X		
Insecta	Lepidoptera	Tineoidea		sp.2		X	E
Insecta	Lepidoptera	Tineoidea		sp.3		X	E

Insecta	Neuroptera	Myrmeleontidae		jovem	X	X	E
Insecta	Psocoptera	Psyllipsocidae		jovem		X	E
Gastropoda	Pulmonata	Subulinidae		sp.3		X	E
Gastropoda	Pulmonata	Systrophiidae	*Happia*	sp.	X		
Malacostraca	Isopoda	Armadillidae		sp.1		X	E
Malacostraca	Isopoda	Philosciidae		sp.2		X	E
Malacostraca	Isopoda	Scleropactidae	*Circoniscus*	*buckupi*	X		
Amphibia	Anura			sp.		4	E/P
Mammalia	Chiroptera	Emballonuridae	*Peropteryx*	sp.		5	E
Mammalia	Chiroptera			sp.	10		E/P
Reptilia	Squamata			sp.	1		E
